# A phase III randomized, open-label, non-inferiority clinical trial comparing liquid and lyophilized formulations of oral live attenuated human rotavirus vaccine (HRV) in Indian infants

**DOI:** 10.1080/21645515.2021.1960136

**Published:** 2021-08-24

**Authors:** Catherine Cohet, Brigitte Cheuvart, Leentje Moerman, Dan Bi, Adrian Caplanusi, Mallesh Kariyappa, Sanjay Lalwani, Monjori Mitra, Amita Sapru, Shruti Saha, P.V. Varughese, Rajeev Zachariah Kompithra, Sanjay Gandhi

**Affiliations:** a GSK Wavre, Belgium; b Department of Pediatrics, Bangalore Medical College & Research Institute, Vani Vilas Women and Children Hospital, Bangalore, India; c Bharati Vidyapeeth Deemed University Hospital, Pune, India; d Department of Pediatrics, Institute of Child Health, Kolkata, India; e Department of Pediatrics, KEM Hospital Research Centre, Pune, India; f Department of Clinical Pharmacology, Seth GS Medical College and KEM Hospital, Mumbai, India; g Department of Pediatrics, Christian Medical College, Ludhiana, India; h Well Baby Immunisation Clinic, Department of Pediatrics, Unit I, Christian Medical College, Vellore, India; i GSK, Mumbai, India

**Keywords:** Rotavirus vaccine, *Rotarix*, liquid, lyophilized, India, immunogenicity, non-inferiority

## Abstract

The human rotavirus vaccine (HRV; *Rotarix*, GSK) is available as liquid (Liq) and lyophilized (Lyo) formulations, but only Lyo HRV is licensed in India. In this phase III, randomized, open-label trial (NCT02141204), healthy Indian infants aged 6–10 weeks received 2 doses (1 month apart) of either Liq HRV or Lyo HRV. Non-inferiority of Liq HRV compared to Lyo HRV was assessed in terms of geometric mean concentrations (GMCs) of anti-RV immunoglobulin A (IgA), 1-month post-second dose (primary objective). Reactogenicity/safety were also evaluated. Seroconversion was defined as anti-RV IgA antibody concentration ≥20 units [U]/mL in initially seronegative infants (anti-RV IgA antibody concentration <20 U/mL) or ≥2-fold increase compared with pre-vaccination concentration in initially seropositive infants. Of the 451 enrolled infants, 381 (189 in Liq HRV and 192 in Lyo HRV group) were included in the per-protocol set. The GMC ratio (Liq HRV/Lyo HRV) was 0.93 (95% confidence interval [CI]: 0.65–1.34), with the lower limit of the 95% CI reaching ≥0.5, the pre-specified statistical margin for non-inferiority. In the Liq HRV and Lyo HRV groups, 42.9% and 44.3% (baseline) and 71.4% and 73.4% (1-month post-second dose) of infants had anti-RV IgA antibody concentration ≥20 U/mL, and overall seroconversion rates were 54.5% and 50.0%. Incidences of solicited and unsolicited adverse events were similar between groups and no vaccine-related serious adverse events were reported. Liq HRV was non-inferior to Lyo HRV in terms of antibody GMCs and showed similar reactogenicity/safety profiles, supporting the use of Liq HRV in Indian infants.

## PLAIN LANGUAGE SUMMARY

What is the context?

- Rotavirus is the most common cause of acute gastronenteritis and contributes to the high number of hospitalizations and deaths in young children worldwide.
- Vaccination against rotavirus has led to a significant decrease in rotavirus-related infections.
- The human rotavirus vaccine *Rotarix* (GSK) is currently used as a liquid or lyophilized formulation.
- In clinical trials conducted in European and North American infants, the liquid vaccine showed ability to induce immune response and safety comparable to the lyophilized formulation.
- Only the lyophilized vaccine is currently marketed in india.

What is new?

- We compared the 2-dose liquid and lyophilized human rotavirus vaccines in indian infants in a phase III clinical trial:
- The ability to induce immune response for thw liquid formulation was not inferior to that observed for the lyophilized vaccine.
- The safety profiles of the 2 formulations were comparable.

Why is this important?

- This study shows that the liquid human rotavirus vaccine can be administrated to infants from india.

## Introduction

Rotavirus (RV) is a prominent cause of acute gastroenteritis, which accounts for substantial morbidity worldwide and persistently high mortality rates in low-income settings.^1^ Despite the significant reduction in the burden of RV disease following worldwide implementation of mass vaccination and further prevention measures, RV continues to disproportionately affect children <5 years of age. In 2016, RV-gastroenteritis resulted in an estimated number of 128,515 deaths worldwide in this age group.^2^

In India, RV morbidity and mortality remain considerable, with around 8% of global RV deaths occurring in this country in 2016.^3^ The Indian Rotavirus Surveillance Network reported that between 2012 and 2014, 35.7–43.0% of children hospitalized with acute gastroenteritis across different regions were RV positive.^4^ More recent etiological studies identified RV as the cause of up to 42.5% of diarrheal disease cases in India and highlighted a great genotypic variety of circulating strains from one region to another and over time.^5–8^

In 2016, India became the first country in the World Health Organization (WHO)’s South Asian region to introduce RV vaccination as part of the national immunization program.^9^ The program was first launched in 9 states,^9^ and reached nationwide implementation in 2019.^10^ Two domestically produced vaccines, the live-attenuated human (nHRV; *Rotavac* liquid, Bharat Biotech International Limited India) and the human-bovine reassortant (BRV-PV; *Rotasiil* lyophilized, Serum Institute of India) vaccines are used in the country’s Universal expanded Immunization Programme (UIP).^9^ The oral live-attenuated human rotavirus vaccine (HRV, *Rotarix* lyophilized, GSK) and the live-attenuated human-bovine reassortant vaccine (HBRV; *RotaTeq* liquid, Merck, United States), the 2 vaccines recommended by the WHO for global use against RV, are also marketed in India.^9^

**Figure 1.**
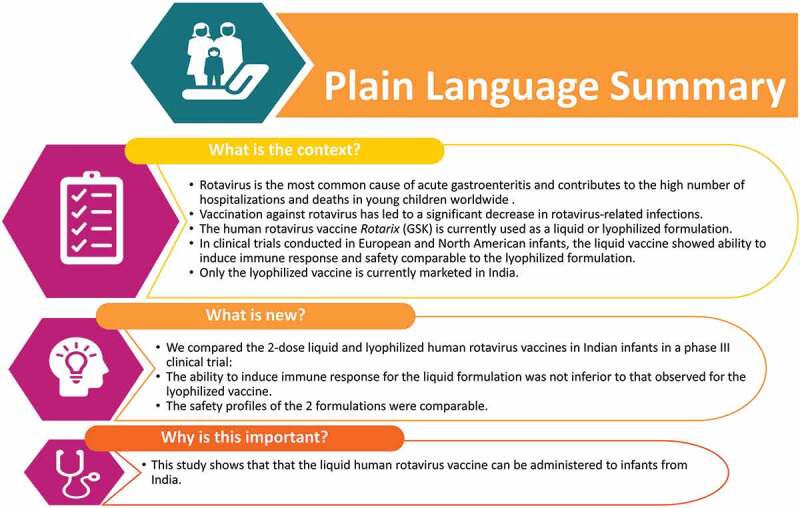
Plain language summary.

HRV is a two-dose vaccine, starting as early as 6 weeks of age and has shown an acceptable safety profile and broad protective efficacy against different RV genotypes, sustained up to the third year of life.^11^ The liquid HRV formulation (Liq HRV) is now the most widely licensed, including in the European Union countries, Canada and Japan. In India, only the lyophilized formulation of the vaccine (Lyo HRV) is licensed, since 2008. Liq HRV was shown to have similar immunogenicity and safety profiles to Lyo HRV^12^ and has the advantage of facilitating storage, handling and administration. The aim of this study was to assess the immunogenicity, reactogenicity and safety of Liq HRV in Indian infants as compared to the licensed Lyo HRV (Figure 1).

## Methods

### Study design and participants

This phase III randomized, open-label, non-inferiority clinical trial was conducted in 8 centers across India from February to December 2019. Healthy infants 6–10 weeks of age at the time of the first vaccination were eligible for enrollment if they had a birth weight >2000 g, and if their parents/legally acceptable representatives were willing and able to comply with protocol requirements, and signed an informed consent form prior to enrollment in the study. Exclusion criteria included planned administration/administration of a vaccine not foreseen by the study protocol in the period starting 30 days before the first dose and ending 30 days after the second dose, with the exception of licensed routine childhood vaccinations as part of local immunization practices and inactivated influenza vaccine, history of confirmed RV-gastroenteritis, history of intussusception, and previous vaccination against RV. A complete list of inclusion and exclusion criteria is available at http://www.gsk-studyregister.com/study/116566.

**Figure 2.**
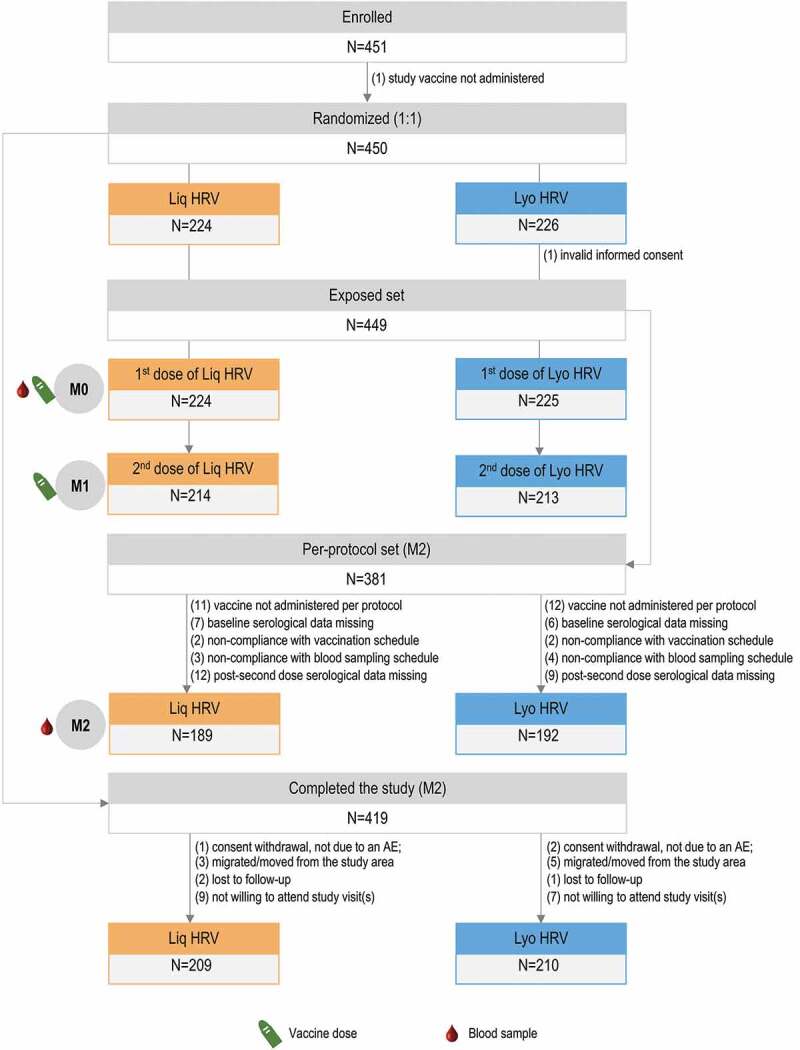
Participant flowchart.

Infants were randomized (1:1) into 2 groups to receive 2 doses of either Liq HRV or Lyo HRV, administered 1 month apart (Figure 2). Randomization was performed with a web-based randomization system, using a minimization procedure accounting for center and the study as factors with equal weight. The study was open label, but laboratory staff responsible for testing were blinded to the intervention.

N, number of infants; Liq HRV, human rotavirus vaccine (liquid formulation); Lyo HRV, human rotavirus vaccine (lyophilized formulation); M, month.

The vaccines were administered orally. An additional dose was allowed in case of regurgitation after vaccination. The vaccines’ compositions have been previously described.^12^

The study was conducted in compliance with the Declaration of Helsinki, the principles of Good Clinical Practice and all applicable regulatory requirements. The study protocol and subsequent amendments were reviewed and approved by the National Regulatory Authority and Institutional Review Boards/Institutional Ethics Committees at each site. The study is registered at www.clinicaltrials.gov (NCT02141204) and www.ctri.nic.in (CTRI/2014/06/004654).

### Study objectives

The primary objective was to demonstrate the non-inferiority of Liq HRV compared to Lyo HRV, in terms of geometric mean concentrations (GMCs) of anti-RV immunoglobulin A (IgA), measured 1-month post-second dose. Non-inferiority was demonstrated if the lower limit (LL) of the 2-sided 95% confidence interval (CI) for the ratio of anti-RV IgA antibody GMCs (Liq HRV over Lyo HRV) was ≥0.5.

Secondary objectives included the assessment of immunogenicity at 1-month post-second dose in terms of seroconversion rates, and evaluation of reactogenicity and safety.

### Immunogenicity assessment

Blood samples (approximately 2 mL) were collected prior to the administration of the first dose and 1-month post-second HRV dose for the determination of anti-RV IgA antibody concentration. Antibody GMCs were calculated pre-vaccination and 1-month post-second HRV dose. Seroconversion rates were calculated post-second dose.

Laboratory testing of the pre-vaccination blood samples from the first 141 infants enrolled in the study indicated that 47.5% of them were seropositive (having anti-RV IgA antibody concentration ≥20 units [U]/mL) prior to vaccination, suggesting an overall high seropositivity rate in the population targeted for enrollment. Therefore, the definition for seroconversion was changed after the study start to allow inclusion of initially seropositive infants in the per protocol set (PPS). Seroconversion was defined as an anti-RV IgA antibody concentration ≥20 U/mL in initially seronegative infants (anti-RV IgA antibody concentration <20 U/mL) or a ≥2-fold increase compared with pre-vaccination values in initially seropositive infants. This definition has been previously used in another HRV clinical trial in Indian infants.^13^ A post-vaccination anti-RV IgA antibody concentration ≥20 U/mL has been previously established as an appropriate correlate of efficacy in HRV clinical trials.^14,15^

Sera were tested at GSK Clinical Laboratories Sciences (Belgium) by a modified enzyme-linked immunosorbent assay used in the clinical development of HRV, as previously described.^16,17^

### Safety and reactogenicity assessment

Adverse events (AEs) were recorded by parents on diary cards. Solicited (cough/runny nose, diarrhea, fever, irritability/fussiness, loss of appetite, and vomiting) and unsolicited AEs were recorded for 8 days (day 1–day 8) and 31 days (day 1–day 31), respectively, following each HRV dose. All AEs were graded on a scale of 1 (mild) to 3 (severe).

Serious AEs (SAEs), as well as AEs/SAEs leading to withdrawal from the study, were assessed throughout the study (starting from dose 1 up to 1-month post-second dose). The causality between each AE/SAE and the study vaccines was assessed by the investigators.

### Statistical analyses

A total number of 450 infants (225 in each study group) was planned for enrollment in order to achieve a target size of at least 292 evaluable infants, assuming that 35% of infants would be non-evaluable/withdrawn (based on a study with Lyo HRV in India).^18^ Assuming identical GMCs in the 2 groups and a 0.79 standard deviation for the log_10_-transformed concentration, the power to reach the primary objective was 90%.

Primary immunogenicity analyses were conducted on the PPS, comprising all eligible infants from the exposed set (ES) who received both doses as per protocol (e.g., compliance to vaccination schedule, no prohibited medication/vaccination administered), complied with the blood sample schedule, and had available immunogenicity data at both sampling timepoints. A supplementary analysis based on the ES was also performed.

Safety analyses were performed on the ES, including all infants that received at least one HRV dose.

GMC calculations were performed by taking the antilog of the mean of the log concentrations transformations; antibody concentrations below the technical cut-off of the assay (13 U/mL) were given a value of half the cut-off. CIs for GMCs were calculated assuming that log-transformed values were normally distributed with unknown variance, by exponential-transformation of the CI for the mean of log-transformed concentration.

The 95% CI for the between-groups anti-RV IgA antibody GMC ratio (primary objective) was estimated using an ANCOVA model on the log-transformed concentrations, including the group and the logarithm of pre-vaccination concentration as covariables. The GMC ratio and 95% 2-sided CI were derived by exponential transformation of the corresponding group contrast in the model. All other comparative analyses were descriptive/exploratory.

Seroconversion rates and the proportion of infants with (S)AEs were calculated with exact 95% CIs. The between-group difference in seroconversion rates was calculated with Miettienen and Manning 95% CIs.^19^

All analyses were performed with the SAS software.

## Results

### Demographics

A total of 449 infants (224 in the Liq HRV group and 225 in the Lyo HRV group) were included in the ES. Two hundred and nine and 210 infants in the Liq HRV and Lyo HRV group, respectively, completed the study. Reasons for not completing are shown in Figure 2. The PPS included 189 infants in the Liq HRV group and 192 in the Lyo HRV group (Figure 2).

The mean age at first dose was 6.8 ± 1.1 weeks in both groups in the PPS and all infants were Asian. The proportion of male infants was slightly higher in the Liq HRV compared to the Lyo HRV group (Table 1). Demographic characteristics in the ES (Supplemental Material, Table S1) were similar to those in the PPS.

**Table 1.** Characteristics of infants (per-protocol set)

	Liq HRV	Lyo HRV
N	189	192
Mean age at first HRV dose (SD), weeks	6.8 (1.1)	6.8 (1.1)
Mean age at second HRV dose (SD), weeks	11.6 (1.3)	11.5 (1.2)
Male, n (%)	106 (56.1%)	91 (47.4%)
Asian ancestry, n (%)	189 (100%)	192 (100%)
Mean height at first HRV dose (SD), cm	55.0 (2.6)	54.8 (2.7)
Mean weight at first HRV dose (SD), cm	4.3 (0.7)	4.3 (0.7)

Liq HRV, human rotavirus vaccine (liquid formulation); Lyo HRV, human rotavirus vaccine (lyophilized formulation); N, number of infants in each group; SD, standard deviation, n (%), number (percentage) of infants in each category.

In total, 85.1% and 82.4% of infants also received routine vaccines at dose 1 and dose 2 of HRV, respectively.

### Immunogenicity

**Figure 3.**
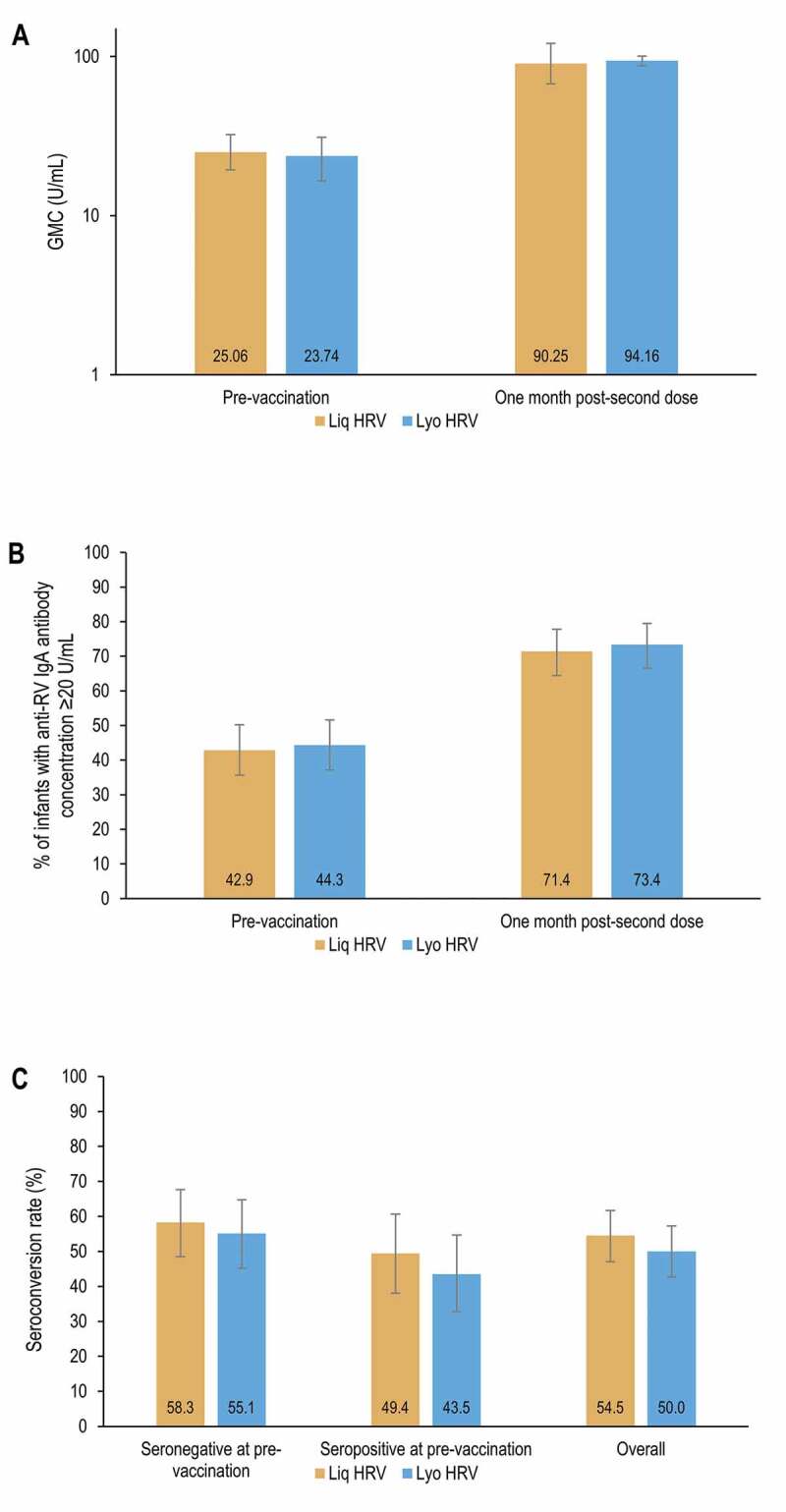
Summary of immunogenicity results: antibody GMCs at pre-vaccination and 1 month post-second dose (A), percentage of infants with anti-RV IgA antibody concentration ≥20 U/mL (B) and seroconversion rates 1 month post-second dose, overall and per pre-vaccination serostatus (C) (per-protocol set).

GMC, geometric mean concentration; RV, rotavirus; IgA, immunoglobulin A; U, units; Liq HRV, human rotavirus vaccine (liquid formulation); Lyo HRV, human rotavirus vaccine (lyophilized formulation). Note: Error bars represent 95% confidence intervals.

At 1-month post-second dose of HRV, the between-group GMC ratio (Liq HRV/Lyo HRV) was 0.93 (95% CI: 0.65–1.34). As the LL of the 95% CI was above the pre-specified statistical margin, non-inferiority of the immunogenicity of Liq HRV compared with Lyo HRV was demonstrated. Similarly, in a supportive analysis conducted only in infants seronegative before vaccination, the LL of the 95% CI for the between-group GMC ratio was 0.52 (Table 2). Anti-RV IgA GMCs were 90.25 U/mL in the Liq HRV group and 94.16 U/mL in the Lyo HRV group (Figure 3A).

**Table 2.** Results of between-group comparison of immunogenicity at 1-month post-dose 2, overall and in infants seronegative at pre-vaccination (per-protocol set)

Group	N	GMC*, U/mL	GMC ratio (Liq HRV over Lyo HRV)
*Non-inferiority of Liq HRV compared to Lyo HRV in terms of GMCs (primary confirmatory objective)*			
Liq HRV	189	88.8	0.93 (95% CI: **0.65**–1.34)
Lyo HRV	192	95.6	
*Between group ratio in infants seronegative at pre-vaccination (secondary, supportive data)*			
Liq HRV	108	43.05	0.88 (95% CI: 0.52–1.49)
Lyo HRV	107	48.97	
Group	N	SC, %	Difference in SC rates (Liq HRV minus Lyo HRV)
*Between-group difference (exploratory analysis)*			
Liq HRV	189	54.5%	4.50 (95% CI: −5.53–14.44)
Lyo HRV	192	50.0%	
*Between-group difference in infants seronegative at pre-vaccination (secondary, supportive data)*			
Liq HRV	108	58.3%	3.19 (95% CI: −10.02–16.30)
Lyo HRV	107	55.1%	

Liq HRV, human rotavirus vaccine (liquid formulation); Lyo HRV, human rotavirus vaccine (lyophilized formulation); N, number of infants in each group; GMC, geometric mean concentration; U, units; CI, confidence interval; SC, seroconversion.

The bolded value indicates that the statistical criteria to demonstrate the confirmative primary objective was met.

* The GMC was estimated from the ANCOVA model for the confirmatory primary objective and the ANOVA model for the secondary, supportive analysis.

At 1-month post-second dose, the difference in seroconversion rates between the Liq HRV group and the Lyo HRV group was lower than 5% in the overall PPS and in infants seronegative at pre-vaccination only (Table 2). The percentages of infants with anti-RV IgA antibody concentration ≥20 U/mL were 71.4% in the Liq HRV group versus 73.4% in the Lyo HRV group. In both groups, seroconversion rates were ≥43.5%, regardless of pre-vaccination serostatus (Figure 3B,C).

The distribution of anti-RV IgA concentrations for both timepoints and groups is presented in Figure S1.

For both groups, immunogenicity results were similar between the ES and the PPS (Table S2).

### Safety and reactogenicity

**Figure 4.**
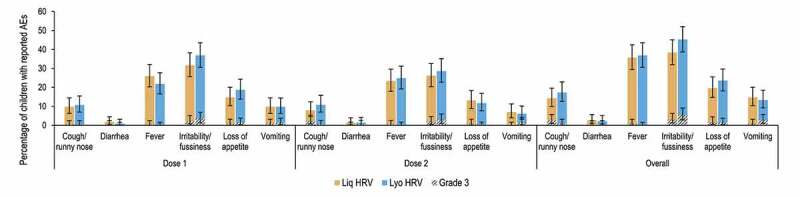
Percentage of infants with reported solicited adverse events occurring within the 8-day period post-vaccination with rotavirus vaccine, after each dose and overall (exposed set).

The most frequently reported solicited AE was irritability/fussiness, which occurred in 38.4% of infants in the Liq HRV group and 45.3% of infants in the Lyo HRV group. Irritability/fussiness was the most common grade 3 solicited AE, reported in 3.1% and 5.3% of infants in the Liq HRV and Lyo HRV groups, respectively. It was also the most commonly reported related solicited AE, in 16.1% of infants in the Liq HRV group and 19.1% of infants in the Lyo HRV group. The most frequent grade 3 related solicited AEs were irritability/fussiness in the Liq HRV group and vomiting in the Lyo HRV group, reported in 1.8% and 2.2% of infants, respectively. Medically-attended solicited general AE were reported in ≤2.7% of infants in both groups, with cough/runny nose being the most common. Figure 4 presents the incidence of solicited general AEs (any and grade 3) following vaccination, after each dose and overall.

Liq HRV, human rotavirus vaccine (liquid formulation); Lyo HRV, human rotavirus vaccine (lyophilized formulation); AE, adverse event. Grade 3 AEs were defined as: “preventing daily activity” for cough/runny nose, “≥6 looser than normal stools/day” for diarrhea, “temperature >39.5°C/103.1°F” for fever (oral, axillar or rectal); “crying that could not be comforted/prevented normal activity” for irritability/fussiness; “did not eat at all” for loss of appetite; “≥3 episodes of vomiting/day” for vomiting. Note: Error bars represent 95% confidence intervals.

Unsolicited AEs were reported in 24.1% and 25.8% of infants receiving Liq HRV and Lyo HRV, respectively, and grade 3 AEs were infrequent (**Table S3**). The most commonly reported unsolicited AEs were injection site swelling in 5.8% and 5.3% of infants, injection site pain in 4.5% and 3.1% of infants, and pyrexia in 4.5% and 5.8% of infants in the Liq HRV and Lyo HRV groups, respectively.

In the Liq HRV group, 9 SAEs (intestinal obstruction, pneumonia, urinary tract infection and respiratory distress each in 1 infant, gastroenteritis in 2 infants, and bronchiolitis in 3 infants) were reported in 7 (3.1%) infants. The intestinal obstruction was mild in intensity, with the onset at 20 days post-dose 2 and a duration of 4 days. In the Lyo HRV group, 3 SAEs (thrombocytopenia, hemorrhagic diarrhea, and dengue fever) were reported in 2 infants (0.9% of infants). All SAEs were recovered/resolved by the end of the study and none were considered related to vaccination by the investigator (**Table S3**).

## Discussion

This is the first study comparing Liq HRV and Lyo HRV in India. The liquid formulation of HRV was developed in response to recommendations from several organizations (WHO, United Nations Children’s Fund [UNICEF]),^12^ encouraging its use due to simplicity of administration, reduction in shipment and storage costs, and increased manufacturing capacity^20^ compared with a lyophilized formulation.

In this study, non-inferiority of immune responses elicited by Liq HRV compared to Lyo HRV was demonstrated in Indian infants, in terms of anti-RV IgA antibody GMCs, at 1 month after completion of the 2-dose schedule. The results are in line with previous reports showing similar immunogenicity and safety profiles for the 2 formulations.^21,22^ In a previous study in 1274 infants aged 6–12 weeks conducted in Panama, in which HRV was co-administered with routine pediatric vaccines, seroconversion rates and anti-RV IgA antibody GMCs were similar between infants receiving the Liq and Lyo HRV formulations.^21^ In another trial, Liq HRV was co-administered with routine vaccines following the WHO’s Expanded Programme on Immunization (EPI) schedule to 750 infants from Vietnam and the Philippines and showed adequate immune responses 1 month-post-vaccination; the results also indicated some flexibility in the vaccination schedule, with similar immunogenicity and safety observed when Liq HRV was administered 1 or 2 months apart.^22^ Moreover, immune responses elicited by both Liq HRV and Lyo HRV in the present study were in the range of those reported for HRV in infants from low- and middle-income countries,^23^ including India.^18^

Data in the current study were generated using a more inclusive definition for seroconversion compared to the one used in most clinical trials with HRV, in which seroconversion was defined as anti-RV IgA concentration ≥20 U/mL post-vaccination in infants with a concentration <20 U/mL pre-vaccination. In both groups in the current study, the observed seroconversion rates obtained with the more inclusive definition were ≥50.0% versus ≥55.1% when assessed with the definition considering only infants seronegative at baseline. The latter value is in line with results obtained in a previous study with Lyo HRV administered according to the same schedule in 363 Indian infants (58.3%), using the earlier definition which excluded initially seropositive infants.^18^ Anti-RV IgA antibody GMCs are also comparable between the 2 trials when excluding initially seropositive infants (49.2 U/mL versus 43.05 and 48.97 U/mL in the current study, in infants receiving Liq HRV and Lyo HRV, respectively). In the current study, the additional criteria to define seroconversion were used to address high seropositivity levels at baseline, and therefore allowed the evaluation of Liq HRV in a population which seems to better reflect the pre-RV vaccination serostatus of infants in India. Indeed, more than 40% of infants in this study showed anti-RV IgA concentrations ≥20 U/mL before vaccination. In another clinical trial in South Indian infants, conducted between March and December 2012, more than half of the infants showed baseline anti-RV IgA concentrations ≥20 U/mL.^13^ The proportion of seropositive infants observed reinforces previous observations of high circulation of RV strains in India,^5–8^ and the mounting of response upon exposure to the pathogen in the first weeks of life.

Safety data generated in this study further support previous evidence that 2 doses of Lyo HRV are well tolerated in infants worldwide,^12,22,24^ including in India.^18,25,26^ In this study, no intussusception cases were reported following administration of HRV, but one infant who received Liq HRV experienced intestinal obstruction, which was resolved within 4 days from onset. No safety concern was observed for either Liq or Lyo HRV administration during the current study, in line with the favorable benefit/risk profile established for RV vaccines.^24^

More than 80% of infants in this study received the HRV doses co-administered with other routine vaccines. Co-administration of Liq HRV with routine pediatric vaccines was previously shown not to impact immune responses or the safety profile of the vaccines in infants in Vietnam and the Philippines.^22^ These data support the use of Liq HRV within the EPI. Currently, nHRV and to a lesser extent BRV-PV are used in the Indian UIP, while HRV and HBRV are available on the Indian private market, allowing access of Indian infants to additional RV vaccines. Of note, between 2012 and 2015, private sector vaccination accounted for 3.4% of RV immunizations in India,^27^ although this contribution has decreased following the increase in coverage under the UIP.

This study’s main strength was the more inclusive definition used for seroconversion, which allows to reflect more accurately the real-world use of the vaccine in a heterogeneous population in terms of exposure to circulating RV. The study also has some limitations. First, the use of different definitions for seroconversion across clinical trials and RV vaccines hinders comparisons with previously published results, including those with HRV. However, the additional criteria were needed to include the high proportion of baseline-seropositive infants and exploratory analyses were also conducted in seronegative infants only, confirming the overall study outcome. Second, the majority of the infants participating in the study were located in Western and Southern India, potentially hampering representativeness of the study population and interpretation of the results, given that the incidence of RV infection varies between regions.^5–8^ In addition, the difference between the ES and PPS was >15%, although analyses showed comparable immunogenicity results between the 2 sets.

## Conclusion

Immune responses induced by Liq HRV were non-inferior to those elicited by Lyo HRV when administered according to a 2-dose schedule in infants from India. The 2 vaccine formulations had a similar reactogenicity and safety profile and no safety concerns were identified. The results of this study support the use of Liq HRV in Indian infants.

## Supplementary Material

Supplemental Material

## Data sharing statement

Anonymized individual participant data and study documents can be requested for further research from www.clinicalstudydatarequest.com.

## Trademark statement

*Rotarix* is a trademark owned by or licensed to the GSK group of companies. *RotaTeq* is a trademark of Merck Inc. *Rotavac* is a trademark of Bharat Biotech International Limited India. *Rotasiil* is a trademark owned by Serum Institute of India Pvt. Ltd.

## References

[1] Rotavirus vaccines, WHO position paper - January 2013. Wkly Epidemiol Rec. 2013;88(5):49–64. 23424730

[2] Troeger C, Blacker BF, Khalil IA, Rao PC, Cao S, Zimsen SR, Albertson SB, Stanaway JD, Deshpande A, Abebe Z, et al. Estimates of the global, regional, and national morbidity, mortality, and aetiologies of diarrhoea in 195 countries: a systematic analysis for the Global Burden of Disease Study 2016. Lancet Infect Dis. 2018;18(11):1211–28. doi:10.1016/s1473-3099(18)30362-1. 30243583 PMC6202444

[3] Troeger C, Khalil IA, Rao PC, Cao S, Blacker BF, Ahmed T, Armah G, Bines JE, Brewer TG, Colombara DV, et al. Rotavirus vaccination and the global burden of rotavirus diarrhea among children younger than 5 years. JAMA Pediatr. 2018;172(10):958–65. doi:10.1001/jamapediatrics.2018.1960. 30105384 PMC6233802

[4] Mehendale S, Venkatasubramanian S, Girish Kumar CP, Kang G, Gupte MD, Arora R. Expanded Indian national rotavirus surveillance network in the context of rotavirus vaccine introduction. Indian Pediatr. 2016;53(7):575–81. doi:10.1007/s13312-016-0891-3. 27508533

[5] Shrivastava AK, Reddy NS, Giri S, Sahu PS, Das M, Mohakud NK, Das RR. Burden and molecular epidemiology of rotavirus causing diarrhea among under-five children: a hospital-based study from Eastern India. J Glob Infect Dis. 2019;11(4):147–52. doi:10.4103/jgid.jgid_16_19. 31849435 PMC6906892

[6] Goldar S, Rajbongshi G, Chamuah K, Alam ST, Sharma A. Occurrence of viral gastroenteritis in children below 5 years: a hospital-based study from Assam, India. Indian J Med Microbiol. 2019;37(3):415–17. doi:10.4103/ijmm.IJMM_19_79. 32003342

[7] Giri S, Nair NP, Mathew A, Manohar B, Simon A, Singh T, Suresh Kumar S, Mathew MA, Babji S, Arora R, et al. Rotavirus gastroenteritis in Indian children <5 years hospitalized for diarrhoea, 2012 to 2016. BMC Public Health. 2019;19(1):69. doi:10.1186/s12889-019-6406-0. 30646867 PMC6334384

[8] Chawla-Sarkar M, Banerjee A, Lo M, Mitra S, Okamoto K, Deb A, Dutta S. A decade-long temporal analyses of human group-A rotavirus among children with gastroenteritis: prevaccination scenario in West Bengal, eastern India. J Med Virol. 2020;92(8):1334–42. doi:10.1002/jmv.25712. 32073164

[9] Malik A, Haldar P, Ray A, Shet A, Kapuria B, Bhadana S, Santosham M, Ghosh RS, Steinglass R, Kumar R. Introducing rotavirus vaccine in the Universal Immunization Programme in India: from evidence to policy to implementation. Vaccine. 2019;37(39):5817–24. doi:10.1016/j.vaccine.2019.07.104. 31474519 PMC6996154

[10] International Vaccine Access Center (IVAC), Johns Hopkins Bloomberg School of Public Health. Pneumonia and diarrhea progress report 2020. [accessed 2021 Jan 11]. http://www.jhsph.edu/ivac/wp-content/uploads/2020/11/IVAC_PDPR_2020.pdf.

[11] European Medicines Agency. *Rotarix* SmPC 24/03/2020 EMA EPAR update. 2020. [accessed 2021 Jan 27]. http://www.ema.europa.eu/en/documents/product-information/rotarix-epar-product-information_en.pdf.

[12] Vesikari T, Karvonen A, Bouckenooghe A, Suryakiran PV, Smolenov I, Han HH. Immunogenicity, reactogenicity and safety of the human rotavirus vaccine RIX4414 oral suspension (liquid formulation) in Finnish infants. Vaccine. 2011;29(11):2079–84. doi:10.1016/j.vaccine.2011.01.004. 21238572

[13] Kompithra RZ, Paul A, Manoharan D, Babji S, Sarkar R, Mathew LG, Kang G. Immunogenicity of a three dose and five dose oral human rotavirus vaccine (RIX4414) schedule in south Indian infants. Vaccine. 2014;32(Suppl 1):A129–133. doi:10.1016/j.vaccine.2014.03.002. 25091666

[14] Baker JM, Tate JE, Leon J, Haber MJ, Pitzer VE, Lopman BA. Post-vaccination serum anti-rotavirus immunoglobulin A as a correlate of protection against rotavirus gastroenteritis across settings. J Infect Dis. 2020;222:309–18. doi:10.1093/infdis/jiaa068. 32060525 PMC7323497

[15] Cheuvart B, Neuzil KM, Steele AD, Cunliffe N, Madhi SA, Karkada N, Han HH, Vinals C. Association of serum anti-rotavirus immunoglobulin A antibody seropositivity and protection against severe rotavirus gastroenteritis: analysis of clinical trials of human rotavirus vaccine. Hum Vaccin Immunother. 2014;10(2):505–11. doi:10.4161/hv.27097. 24240068 PMC4185910

[16] Bernstein DI, Sack DA, Rothstein E, Reisinger K, Smith VE, O’Sullivan D, Spriggs DR, Ward RL. Efficacy of live, attenuated, human rotavirus vaccine 89-12 in infants: a randomised placebo-controlled trial. Lancet. 1999;354(9175):287–90. doi:10.1016/s0140-6736(98)12106-2. 10440305

[17] Bernstein DI, Smith VE, Sherwood JR, Schiff GM, Sander DS, DeFeudis D, Spriggs DR, Ward RL. Safety and immunogenicity of live, attenuated human rotavirus vaccine 89-12. Vaccine. 1998;16(4):381–87. doi:10.1016/s0264-410x(97)00210-7. 9607059

[18] Narang A, Bose A, Pandit AN, Dutta P, Kang G, Bhattacharya SK, Datta SK, Suryakiran PV, Delem A, Han HH, et al. Immunogenicity, reactogenicity and safety of human rotavirus vaccine (RIX4414) in Indian infants. Hum Vaccin. 2009;5(6):414–19. doi:10.4161/hv.5.6.8176. 19276664

[19] Miettinen O, Nurminen M. Comparative analysis of two rates. Stat Med. 1985;4(2):213–26. doi:10.1002/sim.4780040211. 4023479

[20] Wiedenmayer KA, Weiss S, Chattopadhyay C, Mukherjee A, Kundu R, Aye R, Tediosi F, Hetzel MW, Tanner M. Simplifying paediatric immunization with a fully liquid DTP-HepB-Hib combination vaccine: evidence from a comparative time-motion study in India. Vaccine. 2009;27(5):655–59. doi:10.1016/j.vaccine.2008.11.045. 19056443

[21] Velasquez A, Dominguez E, Suryakiran P, Delem A, Ortega-Barria E, Han H. Immunogenicity of the oral live attenuated human rotavirus vaccine RIX4414 (*Rotarix™*) oral suspension (liquid formulation) co-administered with childhood vaccinations in Panama. Int J Infect Dis. 2008;12:e148. doi:10.1016/j.ijid.2008.05.368.

[22] Anh DD, Carlos CC, Thiem DV, Hutagalung Y, Gatchalian S, Bock HL, Smolenov I, Suryakiran PV, Han HH. Immunogenicity, reactogenicity and safety of the human rotavirus vaccine RIX4414 (*Rotarix™*) oral suspension (liquid formulation) when co-administered with expanded program on immunization (EPI) vaccines in Vietnam and the Philippines in 2006-2007. Vaccine. 2011;29(11):2029–36. doi:10.1016/j.vaccine.2011.01.018. 21256876

[23] Gruber JF, Gruber LM, Weber RP, Becker-Dreps S, Jonsson Funk M. Rotavirus vaccine schedules and vaccine response among infants in low- and middle-income countries: a systematic review. Open Forum Infect Dis. 2017;4(2):ofx066. doi:10.1093/ofid/ofx066. 28567431 PMC5445722

[24] World Health Organization (WHO).Global advisory committee on vaccine safety, 6-7 December 2017. Wkly Epidemiol Rec. 2018;93(3):17–32.29350500

[25] Ella R, Babji S, Ciarlet M, Blackwelder WC, Vadrevu KM. A randomized, open-labelled, non-inferiority phase 4 clinical trial to evaluate the immunogenicity and safety of the live, attenuated, oral rotavirus vaccine, ROTAVAC(R) in comparison with a licensed rotavirus vaccine in healthy infants. Vaccine. 2019;37(31):4407–13. doi:10.1016/j.vaccine.2019.05.069. 31178377

[26] Rathi N, Desai S, Kawade A, Venkatramanan P, Kundu R, Lalwani SK, Dubey AP, Venkateswara Rao J, Narayanappa D, Ghildiyal R, et al. A phase III open-label, randomized, active controlled clinical study to assess safety, immunogenicity and lot-to-lot consistency of a bovine-human reassortant pentavalent rotavirus vaccine in Indian infants. Vaccine. 2018;36(52):7943–49. doi:10.1016/j.vaccine.2018.11.006. 30420116 PMC6288065

[27] Farooqui HH, Zodpey S. Private sector vaccine share in overall immunization coverage in India: evidence from private sector vaccine utilization data (2012-2015). Indian J Public Health. 2020;64(1):75–78. doi:10.4103/ijph.IJPH_433_18. 32189688

