# Supplementary material for: A phase III randomized, open-label, non-inferiority clinical trial comparing liquid and lyophilized formulations of oral live attenuated human rotavirus vaccine (HRV) in Indian infants
Source: Hum Vaccin Immunother. 2021 Aug 24;17(11):4646–53. doi: 10.1080/21645515.2021.1960136 (PMC8828117; doi:10.1080/21645515.2021.1960136)
Supplement: Supplemental Material [file KHVI_A_1960136_SM6804.docx]

# Supplementary Material

# F**igure S1.** Reverse cumulative curves for anti-RV IgA concentrations (per protocol set)


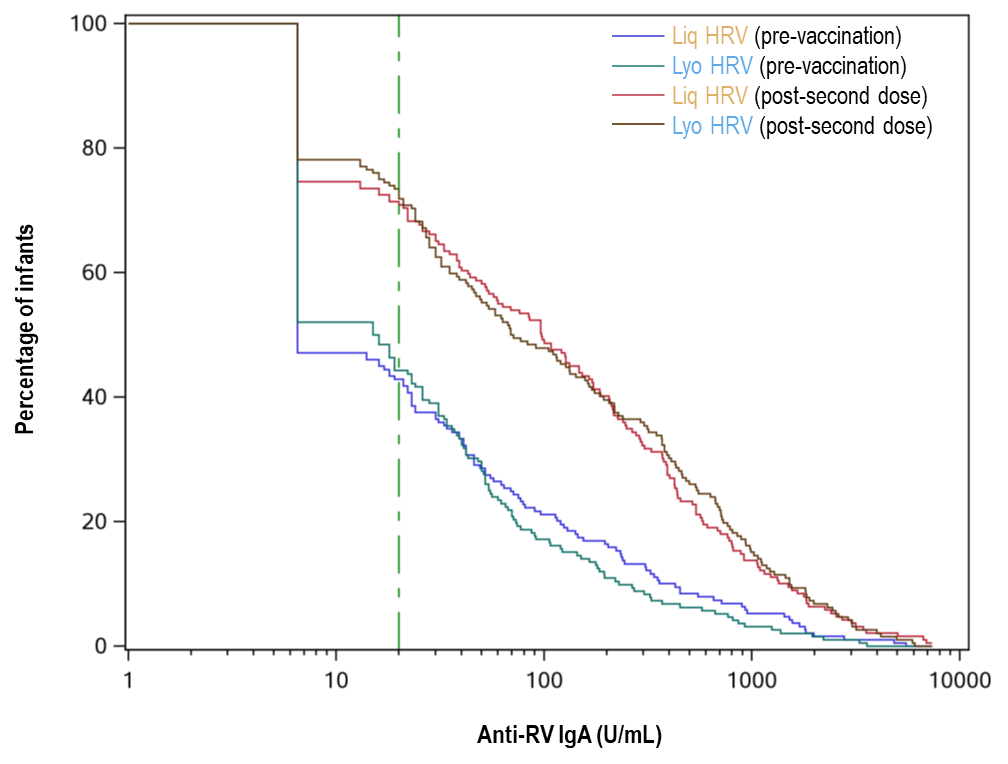


RV, rotavirus; IgA, immunoglobulin A; Liq HRV, human rotavirus vaccine (liquid formulation); Lyo HRV, human rotavirus vaccine (lyophilized formulation); U, units.

Note: The light-green vertical line indicates the threshold for seropositivity (20 U/mL).

## Table S1. Demographic characteristics of infants (exposed set)

|  |  | Liq HRV | Lyo HRV |
| --- | --- | --- | --- |
| N |  | 224 | 225 |
| Infants from each region, n (%) |  |  |  |
| Western India |  | 116 (51.8%) | 118 (52.4%) |
| Southern India |  | 74 (33.0%) | 72 (32.0%) |
| Eastern India |  | 22 (9.8%) | 22 (9.8%) |
| Northern India |  | 12 (5.4%) | 13 (5.8%) |
| Mean age at first HRV dose (SD), weeks |  | 6.8 (1.0) | 6.8 (1.1) |
| Mean age at second HRV dose (SD), weeks |  | 11.6 (1.3) | 11.6 (1.3) |
| Male, n (%) |  | 124 (55.4%) | 105 (46.7%) |
| Asian ancestry, n (%) |  | 224 (100%) | 225 (100%) |
| Mean height at first HRV dose (SD), cm |  | 55.0 (2.6) | 54.8 (2.7) |
| Mean weight at first HRV dose (SD), cm |  | 4.3 (0.7) | 4.3 (0.7) |

Liq HRV, human rotavirus vaccine (liquid formulation); Lyo HRV, human rotavirus vaccine (lyophilized formulation); N, number of infants in each group; SD, standard deviation, n (%), number (percentage) of infants in each category.

## Table S2. Summary of immunogenicity results in the per-protocol and exposed set

|  |  | PPS | | | |  | ES | | | |
| --- | --- | --- | --- | --- | --- | --- | --- | --- | --- | --- |
|  |  | Liq HRV | | Lyo HRV | |  | Liq HRV | | Lyo HRV | |
|  |  | N | value (95% CI) | N | value (95% CI) |  | N | value (95% CI) | N | value (95% CI) |
| % of infants with anti-RV IgA concentration ≥20 U/mL | Pre-vaccination | 189 | 42.9 (35.7–50.2) | 192 | 44.3 (37.1–51.6) |  | 216 | 41.2 (34.6–48.1) | 218 | 44.5 (37.8–51.4) |
|  | Post-second dose | 189 | 71.4 (64.4–77.8) | 192 | 73.4 (66.6–79.5) |  | 201 | 71.1 (64.4–77.3) | 204 | 72.5 (65.9–78.5) |
| Antibody GMC (U/mL) | Pre-vaccination | 189 | 25.06 (19.41–32.35) | 192 | 23.74 (18.96–29.73) |  | 216 | 23.54 (18.71–29.63) | 218 | 24.02 (19.41–29.73) |
|  | Post-second dose | 189 | 90.25 (67.28–121.06) | 192 | 94.16 (70.29–126.13) |  | 201 | 88.20 (66.42–117.13) | 204 | 92.07 (69.35–122.23) |
| Antibody GMC (U/mL) in S+ infants | Pre-vaccination | 81 | 138.24 (99.67–191.73) | 85 | 102.38 (77.58–135.10) |  | 89 | 128.31 (94.45–174.30) | 97 | 103.80 (79.67–135.24) |
|  | Post-second dose | 135 | 248.67 (192.90–320.57) | 141 | 234.21 (179.40–305.76) |  | 143 | 244.89 (191.76–312.74) | 148 | 236.72 (183.07–306.09) |
| Seroconversion rate post-second dose (%) | Overall | 189 | 54.5 (47.1–61.7) | 192 | 50.0 (42.7–57.3) |  | 195 | 54.4 (47.1–61.5) | 198 | 50.0 (42.8–57.2) |
|  | S- infants at pre-vaccination | 108 | 58.3 (48.5–67.7) | 107 | 55.1 (45.2–64.8) |  | 112 | 58.0 (48.3–67.3) | 111 | 55.9 (46.1–65.3) |
|  | S+ infants at pre-vaccination | 81 | 49.4 (38.1–60.7) | 85 | 43.5 (32.8–54.7) |  | 83 | 49.4 (38.2–60.6) | 87 | 42.5 (32.0–53.6) |

PPS, per-protocol set; ES, exposed set; N, number of infants with available serological data in each group and set; Liq HRV, human rotavirus vaccine (liquid formulation); Lyo HRV, human rotavirus vaccine (lyophilized formulation); CI, confidence interval; RV, rotavirus; IgA, immunoglobulin A; GMC, geometric mean concentration; U, units; S+, seropositive (anti-RV IgA concentration ≥20 U/mL); S-, seronegative (anti-RV IgA concentration <20 U/mL).

## Table S3. Percentage of infants with unsolicited adverse events occurring within the 31-day post-vaccination period and serious adverse events occurring up to study end (exposed set)

|  | Liq HRV (N=224) | |  | Lyo HRV (N=225) | |
| --- | --- | --- | --- | --- | --- |
|  | n | % (95% CI) |  | n | % (95% CI) |
| Any unsolicited adverse event | 54 | 24.1 (18.7–30.3) |  | 58 | 25.8 (20.2–32.0) |
| Grade 3 | 6 | 2.7 (1.0–5.7) |  | 3 | 1.3 (0.3–3.8) |
| Related to vaccination* | 0 | 0.0 (0.0–1.6) |  | 0 | 0.0 (0.0–1.6) |
| Medically-attended | 24 | 10.7 (7.0–15.5) |  | 29 | 12.9 (8.8–18.0) |
| Serious adverse events | 7 | 3.1 |  | 2 | 0.9 |
| Related to vaccination* | 0 | 0.0 |  | 0 | 0.0 |
| Fatal | 0 | 0.0 |  | 0 | 0.0 |

Liq HRV, human rotavirus vaccine (liquid formulation); Lyo HRV, human rotavirus vaccine (lyophilized formulation); N, number of infants in each group; n (%), number (percentage) of infants with reported adverse event; CI, confidence interval.

Note: * As assessed by the investigator.

Grade 3 adverse events were defined as “preventing normal, everyday activity”
